# Effectiveness of Finite Element Modelling in Maxillofacial Surgery: A Review of Evidence from Saudi Arabia

**DOI:** 10.7759/cureus.99790

**Published:** 2025-12-21

**Authors:** Mohammed Allahyani, Meshari A Alghamdi, Soltan Alzahrani, Khalid Al Zahrani, Ahmed Alghamdi, Khalid Alghamdi, Ahmed M Alghamdi, Muhannad Alghamdi, Ahmed S Alghamdi

**Affiliations:** 1 Department of Maxillofacial Surgery, King Abdullah Medical City, Makkah, SAU; 2 College of Dentistry, Al Baha University, Al Baha, SAU; 3 Department of General Dentistry, Saudi Commission for Health Specialties (SCFHS), Taif, SAU

**Keywords:** biomechanics, finite element analysis, maxillofacial surgery, methodological quality, saudi arabia, systematic review

## Abstract

Finite Element Analysis (FEA) is a biomechanical simulation tool in surgical planning, but its adoption and methodological quality vary geographically, as a focused analysis of its application in Saudi Arabia is lacking. This review synthesized and evaluated the methodological rigor, biomechanical insights, and translational relevance of Saudi-based studies employing FEA in maxillofacial surgery. A systematic review was conducted following Preferred Reporting Items for Systematic Reviews and Meta-Analyses (PRISMA) guidelines and a registered International Prospective Register of Systematic Reviews (PROSPERO) protocol. Databases were searched for studies with affiliation to Saudi Arabia. Methodological quality and risk of bias were assessed using a bespoke tool based on the FEA best practice. Certainty of evidence was evaluated using a modified Grading of Recommendations Assessment, Development, and Evaluation (GRADE) approach. Three eligible studies covered diverse applications, including mandibular reconstruction, orthognathic surgery, and cranioplasty. Quality assessment revealed methodological heterogeneity, as all studies used patient-specific or anatomically accurate models, but critical best practices have been inconsistently applied. Only one study reported a mesh convergence analysis, while none of the studies performed an experimental validation of their FEA biomechanical predictions. The overall risk of bias was rated moderate to high. Certainty of evidence for any biomechanical outcome was very low according to GRADE. Saudi-based FEA research on maxillofacial surgery is sparse and methodologically nascent. Although technical capabilities in model generation exist, critical gaps in numerical verification (mesh convergence) and experimental validation limit the reliability and translational potential of the findings, underscoring the need to establish standardized, high-quality research protocols to advance data-driven surgical planning in Saudi Arabia.

## Introduction and background

Modern surgical planning is shifting from experience-based approaches to data-driven, personalized medicine [1], a transition that incorporates biomechanical principles to predict clinical outcomes, which is crucial in craniomaxillofacial (CMF) surgery because the skeletal framework is subjected to complex loads during physiological functions, such as mastication and speech [2]. Therefore, a quantitative understanding of the stress and strain distributions within anatomical structures and reconstructive hardware is vital for anticipating postoperative stability, optimizing fixation strategies, and mitigating the risk of mechanical failure. Finite Element Analysis (FEA) is a computational tool that discretizes geometries into a mesh of simpler elements to predict the mechanical response of these structures to applied forces, and it provides an in silico platform for simulating intricate biomechanical scenarios that are difficult or impossible to replicate experimentally [3, 4].

The application of FEA in CMF surgery is extensive, encompassing the biomechanical evaluation of trauma fixation techniques for condylar and mandibular body fractures [5,6], presurgical planning of orthognathic procedures such as Le Fort I osteotomies and mandibular setbacks [3,7,8], and design and assessment of dental implants and major reconstructive hardware [9-11]. FEA is suited to simulate the biomechanical environment of the mandible, a structure subjected to functional loads, thereby offering critical insights for reconstructive strategies, such as those involving free fibula flaps [2, 12].

However, the predictive power and clinical reliability of FEA simulations are not intrinsic properties of the method, but they are dependent on the methodological rigor employed in the simulation workflow, with a credible FEA study being built upon the generation of high-fidelity geometric models derived from patient-specific imaging, the assignment of justified and accurate material properties for both bone and implanted hardware, and the application of physiologically relevant boundary and loading conditions that simulate in vivo function [13-15]. The precision with which these material properties are modeled can affect the accuracy of the simulation outputs [16]. In addition, the credibility of the model’s predictions must be established through numerical verification, such as a mesh convergence analysis, to ensure that the results are independent of mesh density, and through validation, which involves comparing simulation outputs against experimental data or established clinical outcomes [7, 16].

Despite the global proliferation of FEA research in CMF surgery and several systematic reviews on specific applications [11,17], a focused, critical appraisal of the evidence originating from the Middle East, particularly from the Kingdom of Saudi Arabia, is absent. This geographical gap is significant, considering Saudi Arabia's rapidly advancing healthcare technology and national strategic goals of Vision 2030, which emphasizes technological innovation and data-driven personalized medicine. Therefore, this systematic review aimed to synthesize and evaluate the methodological rigor, biomechanical insights, and translational relevance of peer-reviewed, Saudi-based studies that have employed FEA in maxillofacial surgery, seeking to establish the current state of regional research, identify methodological gaps, and provide a framework for advancing high-quality, clinically relevant computational biomechanics research within the Kingdom.

## Review

Methods

Protocol and Registration

This systematic review protocol was developed and registered in the International Prospective Register of Systematic Reviews (PROSPERO) under registration number CRD420251177272, and it was conducted and reported in accordance with the Preferred Reporting Items for Systematic Reviews and Meta-Analyses (PRISMA) 2020 statement [18].

Eligibility Criteria

Primary research studies were included based on a predefined Population, Intervention, Comparator, Outcome, Study Design (PICOS) framework, with a specific emphasis on the research context. The population of interest comprised computational models representing human maxillofacial anatomical structures, with the core intervention being the application of FEA to simulate and evaluate the biomechanical response of these structures to surgical procedures or hardware implantation.

The comparators consisted of alternative surgical techniques, hardware designs, or materials evaluated within the same study. Primary outcomes included quantitative biomechanical data, such as stress, strain, and displacement, with secondary outcomes focusing on methodological quality metrics. Only primary computational simulation studies were eligible for inclusion, with a critical and defining eligibility criterion that the research must have had a substantive institutional affiliation with the Kingdom of Saudi Arabia and a publication date of January 2011 or later.

Information Sources and Search Strategy

A comprehensive search of databases such as Medical Literature Analysis and Retrieval System Online (MEDLINE; via PubMed), Scopus, and Web of Science was performed to identify relevant studies published since January 1, 2011. The search strategy combined keywords and MeSH terms across computational modelling (e.g., "Finite Element Analysis”, "computer simulation”, "biomechanical"), the clinical domain (e.g., "maxillofacial surgery”, "orthognathic”, "mandible”, "cranioplasty"), and the geographical context (e.g., "Saudi Arabia"), supplemented by screening the reference lists of the included articles and relevant reviews to identify any additional studies. The full Boolean search strings used for each database are detailed in Appendix A.

Study Selection

The study selection process followed a two-stage procedure, as titles and abstracts of all identified records were screened for potential eligibility, and records that did not meet the inclusion criteria were excluded. The full texts of all potentially relevant reports were retrieved and assessed against the full eligibility criteria, and the entire selection process was documented using the PRISMA flow diagram.

Data Extraction

A standardized data extraction form was developed and piloted before data extraction from the included studies, and the extracted information was categorized into three main domains, with the first covering general study characteristics such as author details, publication year, clinical domain, surgical procedure, and FEA software used; the second detailing methodological parameters of the FEA workflow, including geometric model source, material property assignment, mesh characteristics, boundary and loading conditions, and any verification or validation procedures; and the third recording key quantitative and qualitative findings, such as primary biomechanical outcomes and authors' conclusions.

Quality and Risk of Bias Assessment

The methodological quality and risk of bias of each included study were independently assessed by two reviewers using a bespoke appraisal tool, which was specifically developed for biomechanical FEA studies by synthesizing and adapting established guidelines, including the American Society of Mechanical Engineers' standards for verification and validation in computational modelling [19].

The tool evaluated five critical domains: (1) geometric fidelity, assessing the anatomical accuracy of the model; (2) mesh convergence, evaluating the verification process to ensure results were independent of mesh density; (3) material properties, assessing the justification and realism of assigned properties; (4) boundary and loading conditions, evaluating the physiological relevance of the simulated environment; and (5) model validation, assessing the comparison of simulation results against experimental or clinical data. The risk of bias was judged for each domain as "Low”, "Moderate”, or "High" based on predefined criteria, culminating in an overall risk of bias rating for each study. A detailed, item-level breakdown of the risk of bias assessment for each included study is provided in Appendix B.

Synthesis of Results and Certainty Assessment

Because of the significant clinical and methodological heterogeneity observed across the included studies, a quantitative meta-analysis was inappropriate. A narrative synthesis of the extracted data was performed to summarize and compare the characteristics, methodological approaches, and principal findings of the studies. The overall certainty of the evidence for the primary biomechanical outcomes was evaluated using a modified Grading of Recommendations Assessment, Development and Evaluation (GRADE) approach, which was designed for preclinical computational research. The certainty of evidence was initiated at "High" and was downgraded based on serious or very serious concerns across five domains: risk of bias, inconsistency of results, indirectness of the evidence, imprecision, and publication bias [20].

Results

Study Selection

A systematic search of electronic databases and registers initially identified 491 records. After the removal of 130 duplicate entries, 361 unique records were subjected to title and abstract screening, of which 339 records were excluded for not meeting the review's eligibility criteria, leaving 22 potentially relevant articles for which full-text reports were sought.

**Figure 1:**
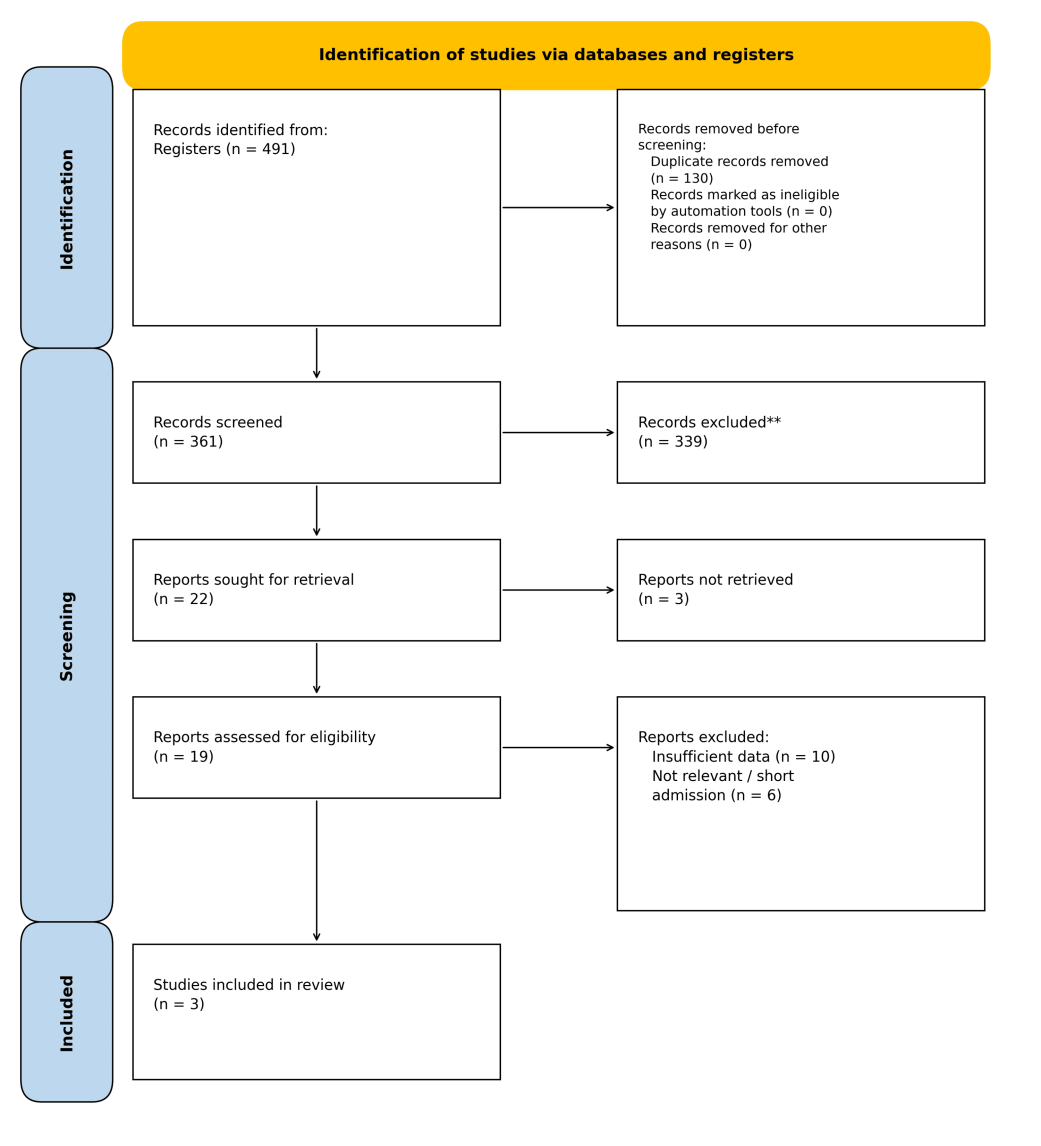
A PRISMA flowchart outlining the study selection process PRISMA: Preferred Reporting Items for Systematic Reviews and Meta-Analyses

Although three of these reports could not be retrieved, the remaining 19 articles underwent full-text eligibility assessment, a comprehensive review that resulted in the exclusion of 16 reports primarily due to insufficient methodological data or a lack of relevance to the review's scope, leading to the inclusion of three primary research studies in the final qualitative and quantitative synthesis, with the entire selection process detailed in the PRISMA flow diagram (Figure 1).

Study Characteristics

The three included studies represent the complete body of peer-reviewed research employing FEA in maxillofacial surgery with substantive institutional affiliation to Saudi Arabia, published since 2011, that met the eligibility criteria. The publication years span from 2015 to 2024, indicating sporadic rather than continuous research output in this domain. All studies were affiliated with major academic institutions within Saudi Arabia, namely King Saud University and Riyadh Elm University (Table 1).

**Table 1:** Summary of characteristics and demographics of included studies FEA: Finite Element Analysis; MIMICS: Materialise Interactive Medical Image Control System; ANSYS: ANalysis SYStem; USSO: unilateral sagittal split osteotomy; PEEK: polyether-ether-ketone

Study	Year	Country of Affiliation	Clinical Domain/Surgical Procedure	FEA Software Used	Study Aim
Al-Ahmari et al. [21]	2015	Saudi Arabia (King Saud University)	Mandibular reconstruction (tumor resection)	MIMICS, 3-Matic, ANSYS Workbench 14	To compare the biomechanical performance of a novel "sinewave" design reconstruction plate against a conventional straight plate.
Assari et al. [22]	2024	Saudi Arabia (Riyadh Elm University, Maleen Consultant Center)	Orthognathic surgery (USSO)	MIMICS, SOLIDWORKS, Hypermesh, ANSYS	To assess the biomechanical properties (stress, displacement) of the mandible, condyle, and fixation hardware following a simulated USSO procedure.
Moiduddin et al. [23]	2023	Saudi Arabia (King Saud University)	Cranioplasty/Cranial reconstruction	MIMICS, 3-Matic, Magics, ANSYS 19.1, Hypermesh	To propose and evaluate an efficient workflow for designing, fabricating, and assessing the fitting accuracy and biomechanical stability of a custom porous PEEK cranial implant.

The clinical applications of FEA across the included literature demonstrate a diversity, covering three distinct domains within maxillofacial surgery; specifically, the research encompasses mandibular reconstruction following tumor resection [21], planning for orthognathic surgery via unilateral sagittal split osteotomy (USSO) [22], and the design and assessment of implants for cranioplasty [23]. This heterogeneity extends to the specific aims of each study, which ranged from comparing the biomechanical performance of a novel "sinewave" reconstruction plate against a conventional straight plate to assessing the stress distribution on the mandibular condyle following a simulated osteotomy and evaluating the biomechanical stability and fitting accuracy of a custom porous polyether-ether-ketone (PEEK) cranial implant. The software used for the FEA workflows included industry-standard packages, with Materialise Interactive Medical Image Control System (MIMICS) being a choice for geometric model generation from medical imaging data, and ANalysis SYStem (ANSYS) is the software for conducting the numerical simulations.

Methodological Quality and Risk of Bias

**Figure 2:**
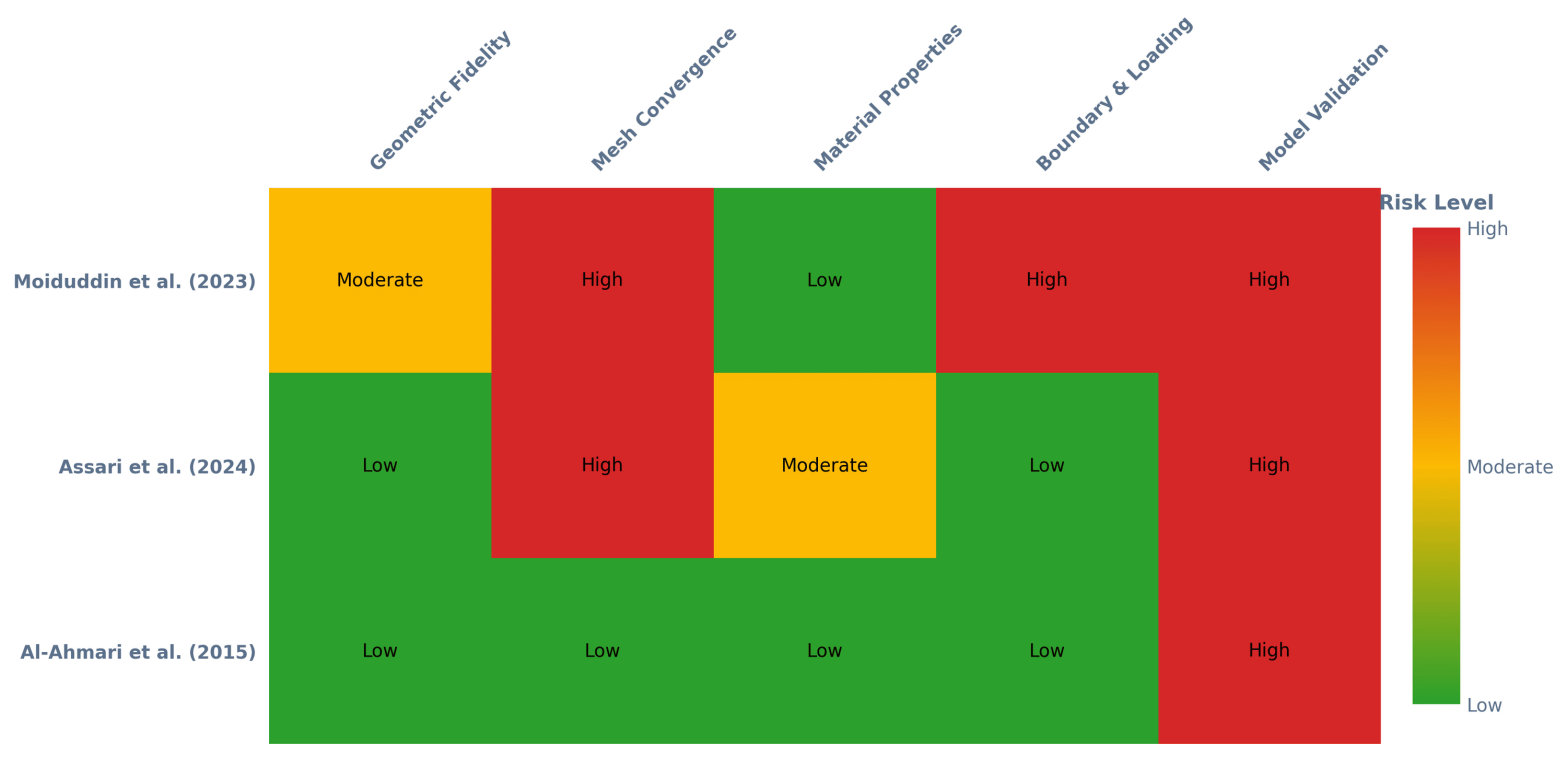
Risk of bias assessment summary The risk of bias assessment for the included studies: Al-Ahmari et al. [21], Assari et al. [22], and Moiduddin et al. [23].

The methodological quality and risk of bias assessment revealed heterogeneity across the three included studies, with common strengths in model generation but critical recurring weaknesses in numerical verification and experimental validation (Figure 2). The overall risk of bias for the body of evidence was judged to range from moderate to high, a determination largely influenced by deficiencies in the latter, more rigorous methodological steps.

A notable strength observed across the evidence base was in the domain of geometric fidelity, as both Al-Ahmari et al. [21] and Assari et al. [22] used patient-specific computed tomography (CT) scans to generate high-fidelity, clinically relevant anatomical models, thereby earning a "low risk" of bias judgment in this domain, while Moiduddin et al. [23] employed an idealized model derived from a "clean skull”, which was appropriate for their objective of evaluating a general design process, but it was rated as "moderate risk" due to its non-patient-specific nature. In addition, the assignment of material properties was generally robust, with two studies differentiating between various tissue types based on cited literature ("low risk") [21,23], while Assari et al. [22] simplified the bone into a single homogenous object, introducing a potential source of inaccuracy and thus a "moderate risk" of bias.

The physiological relevance of the applied boundary and loading conditions varied considerably, as the studies by Al-Ahmari et al. [21] and Assari et al. [22] simulated masticatory muscle forces derived from the literature, representing physiologically relevant scenarios and thus warranting a "low risk" rating, but Moiduddin et al. [23] applied a highly simplified, non-physiological static force intended to simulate the gravitational force of the head, a condition that limits the clinical applicability of the stress analysis and resulted in a "high risk" of bias judgment for this domain.

A critical methodological weakness was identified in the inconsistent application of mesh convergence analysis. Only Al-Ahmari et al. [21] performed and reported a mesh convergence study, which is a fundamental practice for ensuring the numerical reliability of FEA results, thus receiving a "low risk" assessment for this domain. The absence of this verification step in the studies by Assari et al. [22] and Moiduddin et al. [23] introduces a substantial risk that their findings may be dependent on mesh density rather than true biomechanical behavior, leading to a "high" risk of bias judgment.

The most significant and universal methodological deficit across all three studies was the complete lack of experimental model validation for the FEA predictions, as none of the studies compared their computational predictions of stress, strain, or displacement against physical biomechanical testing, such as in vitro experiments with strain gauges. Although Moiduddin et al. [23] performed a geometric validation to quantify the manufacturing accuracy of their implant, this does not constitute a validation of the biomechanical simulation's predictive accuracy, which results in a "high risk" of bias for this domain across the entire evidence base, limiting the confidence in the translational and clinical applicability of the quantitative findings. The overall risk of bias was rated as "moderate" for Al-Ahmari et al. [21] and "high" for both Assari et al. [22] and Moiduddin et al. [23].

Quantitative Findings

**Figure 3:**
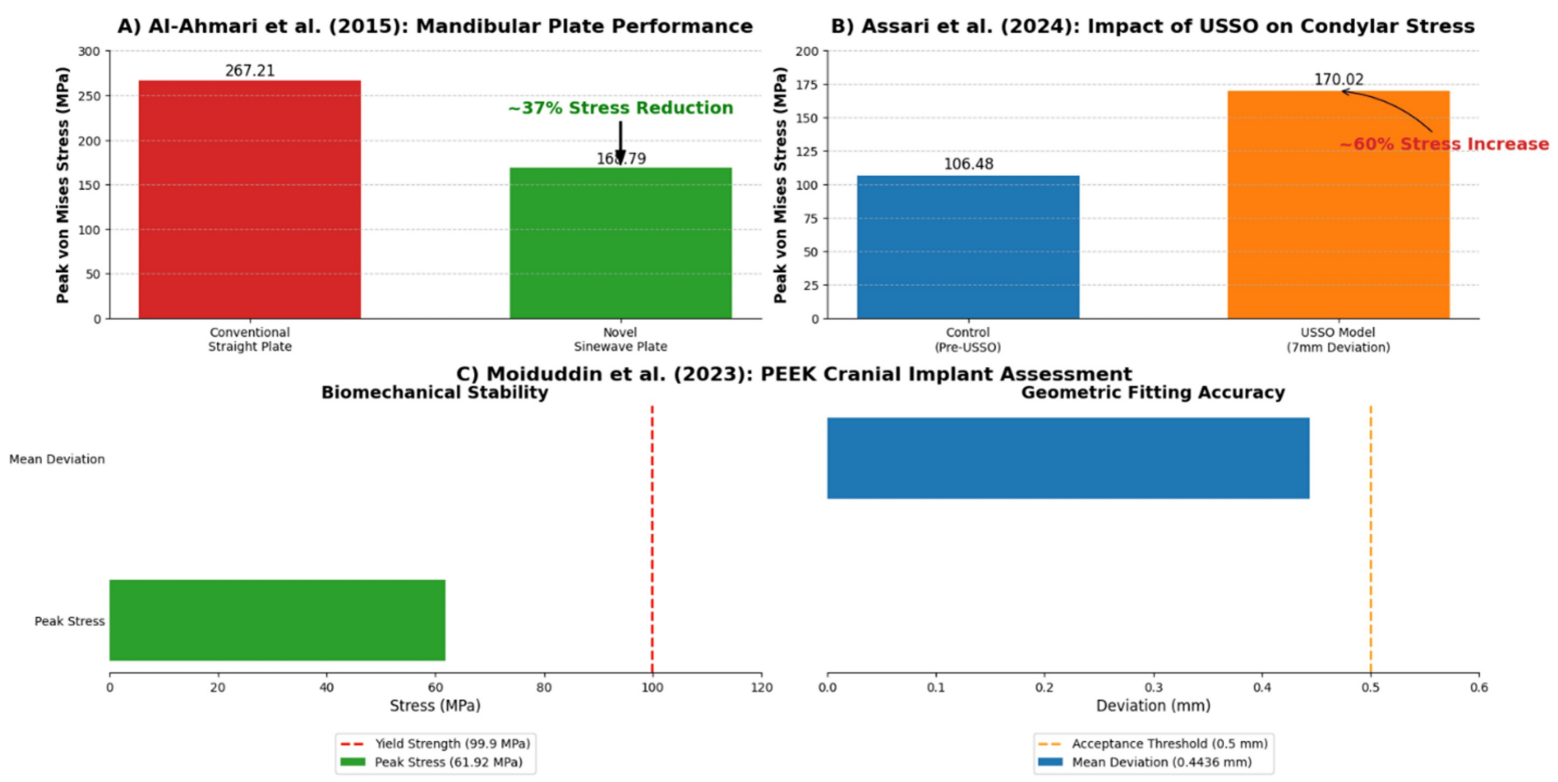
Synthesis of key quantitative findings from the included Saudi-based FEA studies (A) Comparative analysis of von Mises stress on conventional straight plates versus novel "sinewave" plates for mandibular reconstruction [21]. (B) Comparison of peak von Mises stress on the mandibular condyle before and after a simulated unilateral sagittal split osteotomy (USSO) with midline deviation [22]. (C) Assessment of peak stress and geometric fitting deviation for a custom porous polyether-ether-ketone (PEEK) cranial implant [23]. FEA: Finite Element Analysis

The quantitative data extracted from the three included studies are characterised by significant heterogeneity in both the primary outcomes measured and the magnitude of the reported biomechanical values, which preclude any form of meta-analysis or direct numerical comparison; therefore, the principal quantitative findings from each study are presented individually (Figure 3).

The investigation by Al-Ahmari et al. [21] conducted a comparative analysis of two mandibular reconstruction plate designs (Figure 3A). The study reported that the conventional straight plate design resulted in a peak von Mises stress of 267.21 MPa on the fixation screws under simulated physiological loading, while the novel "sinewave" plate design demonstrated a substantial biomechanical advantage, with a peak stress of 168.79 MPa, which represents a stress reduction of approximately 37%, quantitatively supporting the authors' conclusion that the novel design may enhance the stability of mandibular reconstruction.

Assari et al. [22] focused on quantifying the biomechanical consequences of a USSO procedure on the contralateral mandibular condyle. The findings indicated an increase in stress concentration on the non-operated condyle post procedure; specifically, the peak von Mises stress in the condylar region increased from a baseline of 106.48 MPa in the preoperative control model to 170.02 MPa in the postoperative model that simulated a 7 mm midline deviation, which corresponds to an approximately 60% increase in localized stress on the condyle (Figure 3B), highlighting a critical biomechanical trade-off of the USSO technique.

Moiduddin et al. [23] reported two distinct quantitative outcomes related to their custom porous PEEK cranial implant, one pertaining to biomechanical stability and the other to geometric fitting accuracy. The FEA component of the study demonstrated that under a simplified static load of 50 N, the implant experienced a peak von Mises stress of 61.92 MPa, which is well within the material's reported yield strength of 99.9 MPa, indicating that the implant is biomechanically stable under the tested conditions. The geometric validation of the manufactured implant yielded a mean fitting deviation of 0.4436 mm compared to the original digital design. This measure of manufacturing precision is below the clinical acceptance threshold of 0.5 mm, confirming a high degree of geometric accuracy for the fabrication process (Figure 3C).

Certainty of Evidence

The overall certainty of the body of evidence regarding the biomechanical performance of maxillofacial interventions derived from Saudi-based FEA studies was evaluated using a modified GRADE approach designed for preclinical computational research. The final assessment concluded that the certainty of the evidence for any biomechanical outcome was very low (Table 2).

**Table 2:** GRADE summary of dindings for biomechanical outcomes GRADE: Grading of Recommendations Assessment, Development, and Evaluation

No. of Studies	Study Design	Risk of Bias	Inconsistency	Indirectness	Imprecision	Final Certainty
3	Non-randomized computational simulations	Serious	Very serious	Serious	Very serious	⊕◯◯◯ Very low

The certainty of evidence was downgraded from high to moderate due to a serious risk of bias. This was driven by critical methodological omissions in most studies (two of three), most notably the absence of a reported mesh convergence analysis, which weakens confidence in the numerical reliability of the findings.

A second downgrade, from moderate to low, was needed due to serious concerns for indirectness. All included studies were in silico simulations and thus indirect representations of clinical reality, but this indirectness was critically amplified by the absence of experimental or clinical validation. Without comparison to physical testing, the predictive accuracy of the models remains unverified.

The final downgrade to very low was required due to very serious inconsistency across the evidence base. The included studies are clinically and methodologically heterogeneous, each investigating a distinct scenario: mandibular reconstruction plates, USSO, and cranial PEEK implants. This divergence in clinical application, models, and methods prevented any meaningful comparison or synthesis of effects.

Although serious concerns regarding imprecision were also noted, since the entire evidence base was composed of only three individual computational studies analogous to clinical case reports, no further downgrading was possible because the certainty level was already at its lowest grade. Finally, while publication bias is suspected, as is common in computational literature where studies with successful or significant findings are more likely to be published, it was not possible to formally assess this with such a limited number of studies, as after sequential downgrading for serious concerns related to risk of bias, indirectness, and inconsistency, the overall certainty of the current body of Saudi-based evidence is judged to be very low.

Discussion

Summary of Principal Findings

This systematic review revealed that the body of peer-reviewed, Saudi-based literature employing FEA in maxillofacial surgery is nascent, sparse, and methodologically heterogeneous. The synthesis of the three identified studies indicates a foundational technical capability in generating high-fidelity, patient-specific geometric models from clinical imaging data; however, this capability is undermined by critical and recurrent methodological shortcomings. The most salient of these deficiencies include the inconsistent application of numerical verification through mesh convergence analysis and an absence of experimental validation for biomechanical predictions. Individual studies demonstrate technical promise in specific applications such as the comparative design of reconstruction plates or the assessment of postoperative stress on the mandible, but the evidence base is not robust enough to support definitive clinical conclusions or guide surgical practice. The overall risk of bias across the evidence was high, precluding a confident appraisal of the translational value of the current research.

Interpretation of Findings in a Global Context

The methodological patterns observed in Saudi-based literature are not unique, although certain deficits appear acute. The absence of experimental validation, identified as a weakness in this review, represents a validation gap that challenges the FEA field [16]. The comparison of in silico predictions with in vitro or in vivo data is a resource-intensive but essential step for establishing the predictive credibility of a computational model. The absence of this step in the regional sample suggests a lag in adopting more rigorous, multidisciplinary research paradigms that integrate computational and experimental work, which are considered the gold standard for generating high-fidelity predictive models [6].

Similarly, the inconsistent reporting of mesh convergence analysis represents a verification gap, as this step is a component of computational best practice, ensuring that the numerical solution is independent of mesh discretization, and its omission in two of the three studies is a serious methodological flaw that distances this portion of the research from established international standards of rigor.

Strengths

The principal strength of this review is its novelty as the first systematic synthesis of FEA research in maxillofacial surgery conducted within Saudi Arabia, guided by a prospectively registered protocol and employing a rigorous bespoke tool for quality and risk of bias assessment, which provides a transparent and robust baseline for understanding the state of the field in Saudi Arabia.

Limitations

The review is constrained by the sparsity of the literature it sought to analyze. The inclusion of only three studies precludes any quantitative meta-analysis and limits the ability to draw definitive conclusions about temporal or methodological trends. In addition, the search strategy was comprehensive for peer-reviewed literature, but there remains a potential for having missed grey literature such as unpublished institutional reports or conference proceedings, which might contain additional relevant data but were not indexed in the searched databases.

Recommendations for Future Research in Saudi Arabia

Based on the research gaps and methodological shortcomings, a clear and actionable framework is necessary to advance the quality and clinical impact of FEA research in maxillofacial surgery within Saudi Arabia; therefore, this review proposes recommendations for building high-quality, regionally relevant evidence that can support clinical innovation and align with national healthcare goals.

Future studies must prioritize and incorporate experimental validation as a standard component of the research workflow, as comparing simulation predictions with data from physical models, even simplified ones, is essential for quantifying model accuracy and building the clinical confidence required for translational applications. Standardized reporting is imperative for enhancing reproducibility and comparability across studies, requiring the advocacy for adherence to established guidelines for reporting FEA studies, with the inclusion of a mesh convergence analysis to ensure and demonstrate the numerical reliability of the computational results.

Enhancing multidisciplinary collaboration among clinicians, surgeons, and biomedical engineers is essential to ensure that computational models are methodologically robust and clinically relevant, addressing surgical questions with physiologically appropriate simulations. Future research should target specific, under-researched clinical areas identified in the global literature, where biomechanical investigations into the stability of different orthognathic movements beyond simple advancements, the challenges of pediatric CMF applications, or comparative analyses of novel biomaterials for reconstruction [2] represent opportunities for generating high-impact, regionally relevant research.

## Conclusions

The current body of Saudi-based FEA research in maxillofacial surgery is nascent and methodologically inconsistent, preventing any definitive conclusions on its clinical effectiveness. Despite evidence of technical capability in generating high-fidelity models, critical methodological gaps, particularly the absence of numerical verification and experimental validation, were pervasive. Therefore, establishing standardized, high-quality research protocols that mandate both mesh convergence analysis and experimental validation is imperative to advance data-driven surgical innovation in the Kingdom and align with its national healthcare and technology goals.

## Appendices

Appendix A 

**Table 3:** Full search strategy

Search Component	MeSH Terms and Keywords Used	Syntax
FEA Concepts	MeSH Terms: "Finite Element Analysis" Keywords: "Finite Element Analysis", "Finite Element Model*", "Finite Element Method*", "FEA", "FEM", "Computer Simulation", "Computational Model*", "Biomechanical Analysis", "In Silico"	("Finite Element Analysis"[Mesh] OR "Finite Element Analysis"[tiab] OR "Finite Element Model*"[tiab] OR "Finite Element Method*"[tiab] OR "FEA"[tiab] OR "FEM"[tiab] OR "Computer Simulation"[tiab] OR "Computational Model*"[tiab] OR "Biomechanical Analysis"[tiab] OR "In Silico"[tiab])
CMF Domain	MeSH Terms: "Maxillofacial Surgery", "Mandible", "Maxilla", "Orthognathic Surgery", "Cranioplasty" Keywords: "Maxillofacial", "Craniofacial", "Orthognathic", "Mandible", "Mandibular", "Maxilla", "Maxillary", "Cranioplasty", "Le Fort", "Sagittal Split", "Osteotomy", "Reconstruction Plate", "Dental Implant*", "Zygoma"	("Maxillofacial Surgery"[Mesh] OR "Mandible"[Mesh] OR "Maxilla"[Mesh] OR "Orthognathic Surgery"[Mesh] OR "Cranioplasty"[Mesh] OR "Maxillofacial"[tiab] OR "Craniofacial"[tiab] OR "Orthognathic"[tiab] OR "Mandible"[tiab] OR "Mandibular"[tiab] OR "Maxilla"[tiab] OR "Maxillary"[tiab] OR "Cranioplasty"[tiab] OR "Le Fort"[tiab] OR "Sagittal Split"[tiab] OR "Osteotomy"[tiab] OR "Reconstruction Plate"[tiab] OR "Dental Implant*"[tiab] OR "Zygoma"[tiab])
Geographic	MeSH Term: "Saudi Arabia" Keywords: "Saudi Arabia", "KSA", "Saudi"	("Saudi Arabia"[Mesh] OR "Saudi Arabia"[Affiliation] OR "KSA"[Affiliation] OR "Saudi"[Affiliation])
Final Query	Combines the three components with the AND operator.	

Appendix B 

**Table 4:** Item-level risk of bias assessment

Study ID & Citation	Domain	Judgement	Justification for Judgement
1. Al-Ahmari et al. [21]	Geometric Fidelity	Low Risk	Patient-specific model derived from a relevant clinical case (jaw tumor CT). Clear segmentation of cortical and cancellous bone.
	Mesh Convergence	Low Risk	Best Practice: A mesh convergence (sensitivity) study was explicitly performed and reported with a graph, ensuring results are independent of mesh density.
	Material Properties	Low Risk	Differentiated properties for all components (cortical/cancellous bone, implant) were used and cited from appropriate literature.
	Boundary & Loading	Low Risk	Physiologically relevant loading conditions simulating masticatory muscle forces were applied based on literature. Load sensitivity was also tested.
	Model Validation	High Risk	Critical Omission: The study is purely computational. No comparison of FEA results against any in vitro experimental data or clinical outcomes was performed.
2. Assari et al. [22]	Geometric Fidelity	Low Risk	Patient-specific model derived from a CT scan of a human mandible, representing appropriate anatomical accuracy.
	Mesh Convergence	High Risk	Critical Omission: The authors did not report performing a mesh convergence or sensitivity analysis. The reliability and stability of the numerical solution are therefore unverified.
	Material Properties	Moderate Risk	Bone was simplified as a single homogenous object, not differentiating between cortical and cancellous bone. This simplification can affect stress distribution accuracy.
	Boundary & Loading	Low Risk	Physiologically relevant loading conditions simulating both muscle and occlusal forces were applied based on literature.
	Model Validation	High Risk	Critical Omission: The study is purely in silico. No experimental validation was performed to confirm the predictive accuracy of the stress predictions.
3. Moiduddin et al. [23]	Geometric Fidelity	Moderate Risk	An idealized model ("clean skull") was used instead of a patient-specific clinical case. While suitable for a general process evaluation, it reduces direct clinical applicability.
	Mesh Convergence	High Risk	Critical Omission: The authors did not report performing a mesh convergence study. The numerical reliability of the FEA results is unknown.
	Material Properties	Low Risk	Differentiated properties for all components (skull, PEEK implant, titanium screws) were appropriately assigned and cited.
	Boundary & Loading	High Risk	Major Simplification: A single, non-physiological static load (50 N) was applied. This does not represent the complex, dynamic loading environment of the cranium and limits the clinical relevance of the stress analysis.
	Model Validation (FEA)	High Risk	Critical Omission: The biomechanical FEA predictions (stress/strain) were not validated. Note: A separate geometric validation of fitting accuracy was performed and was of high quality, but this does not validate the FEA.
